# National stroke management plan in Uruguay: Challenges and opportunities

**DOI:** 10.3389/fneur.2022.973380

**Published:** 2023-02-01

**Authors:** Ignacio Amorín, Adolfo Savia, Andres Gaye, Claudia Camejo, Brayan Triviño, Matías Muñoz, Sebastian Yancev, Tamara Menendez, Rodrigo Decima

**Affiliations:** ^1^Brain Health Program, Ministry of Public Health of Uruguay, Montevideo, Uruguay; ^2^Hospital San Juan de Dios (San Juan de Dios Hospital), Buenos Aires, Argentina; ^3^Hospital de Clínicas (Clinic Hospital), School of Medicine, Montevideo, Uruguay; ^4^Sociedad Uruguaya de Neurología (Uruguayan Neurology Association), Montevideo, Uruguay; ^5^Sociedad Uruguaya de Emergencistas (Uruguayan Association of Emergency Physicians), Montevideo, Uruguay; ^6^Comisión Honoraria de Salud Cardiovascular (Honorary Commission for Cardiovascular Health), Montevideo, Uruguay

**Keywords:** stroke, public health, Uruguay, care system, thrombolysis

## Abstract

Stroke accounts for 5.5% of the national Global Burden of Disease (GBD) and ~2,000 deaths per year in Uruguay. To respond to this medical emergency, the Ministry of Public Health (MPH) of Uruguay devised the National Stroke Plan (NSP). Scientific associations, universities, scholars, and patient organizations, both at the national and international levels, took part in the process, which ended with the generation of the national stroke management guidelines, including measures based on the best evidence available. This was accompanied by presidential regulatory decrees and several ordinances that set the foundations of the legal framework for their implementation as of 2020. Forty-two Stroke Ready Centers (SRC) and seven Comprehensive Stroke Centers (CSC) were strategically established and interlinked to ensure compliance with international accessibility recommendations, offering, in turn, the required training for their healthcare teams. A pre-hospital care protocol was also created for all countrywide mobile units. For NSP assessment, stroke was included as a “Care Goal (objective)” for the whole health system, providing the involved healthcare organizations with a financial incentive for compliance with the basic objectives related to the treatment of hyper acute stroke. The NSP came into force during the COVID-19 pandemic and, considering the special circumstances imposed, it made it possible to maintain hyper acute medical care and increase population access to recanalization treatment, particularly mechanical thrombectomy. The purpose of this article is to share our experience in the development of the NSP by describing some preliminary outcomes.

## Introduction

Stroke accounts for ~5.5 % of the GBD (1) of Uruguay. It causes around 2,000 deaths per year and is the first leading cause of disability in the country (2). Due to the importance of this condition, in 2020, the MPH of Uruguay established stroke as a top priority and, through the development of a specific plan, it formalized some actions having an impact on the prevention, diagnosis, treatment, and rehabilitation with a clear view to reducing the burden of disease in the population of Uruguay. In this article, we will describe the characteristics of the National Stroke Plan (NSP) of Uruguay: planning, execution, and assessment thereof. As there are different approaches to address stroke from the perspectives of health systems at a global level, we share our experience and some initial outcomes of the NSP to contribute to the development of strategies aimed at reducing morbimortality arising from this condition in Latin America (3).

### Impact of stroke in Uruguay

**Figure 1:**
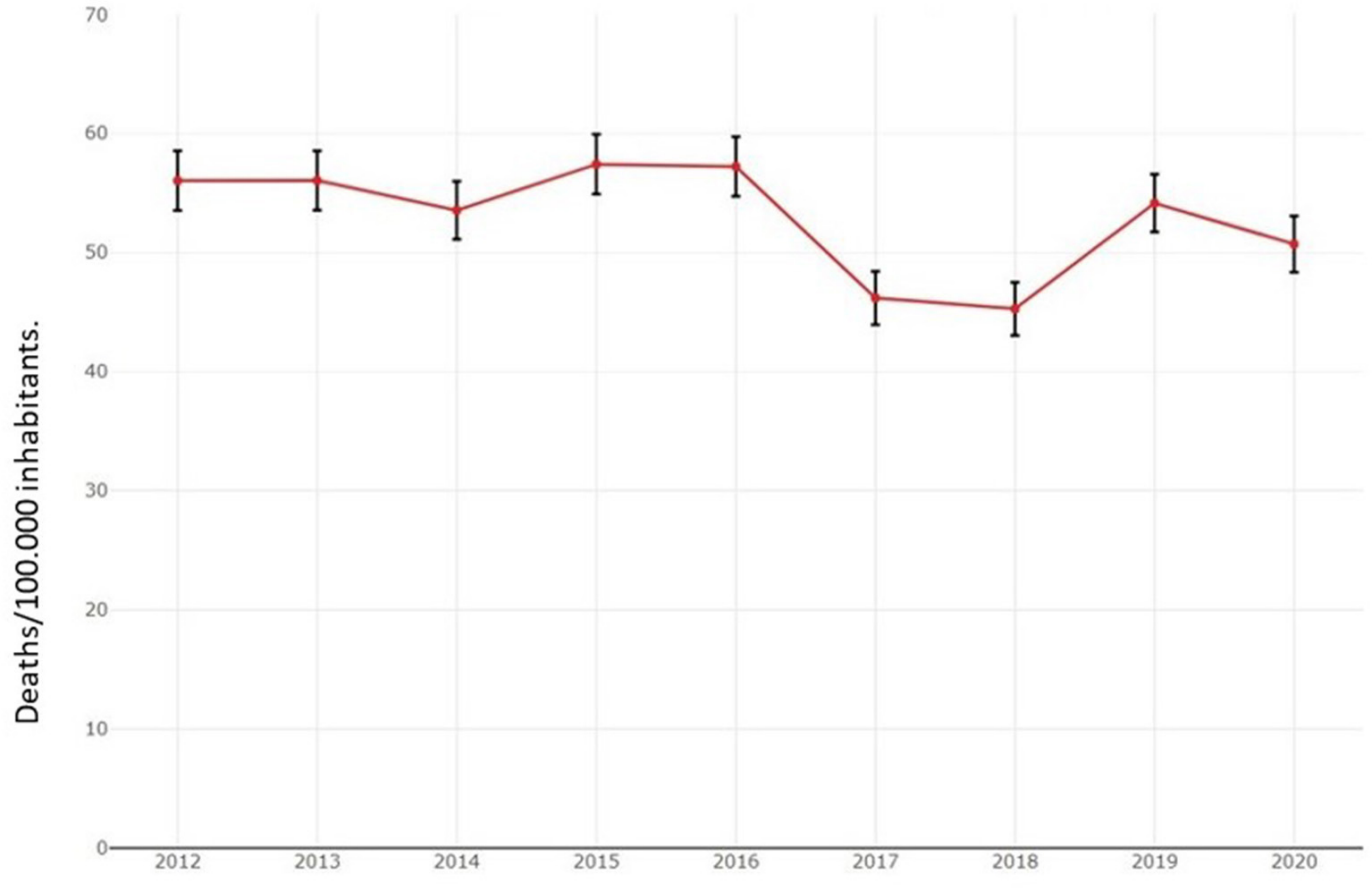
Mortality progress due to stroke in Uruguay from 2012 to 2020.

The average annual incidence of stroke in Uruguay is 125.14/100,000 inhabitants and has remained stable with no significant changes from 2016 to 2020. According to an epidemiological study carried out in the town of Rivera, 73.4 % of the strokes were ischemic (4). There is a slight predominance in female subjects with 51.74 %. The average mortality rate from 2012 to 2020 was 52.9/100.00 inhabitants (in 2019, 1905 deaths were recorded, and in 2020, 1790 were recorded (I61–I64 International Classification of Diseases v10 ICD-10) with a decreasing trend in recent years (Figure 1) (5). The mean age was 72.8 years, and the median age was 75 years (6).

### Characteristics of the health system of Uruguay

Uruguay is a country located in South America, with a total surface of 176,220 km^2^ and coasts on Río de la Plata and the Atlantic Ocean. According to the last 2011 census (7): its population is 3,390,077 with an annual growth rate of 1.9 %. It has a birth rate of 13.62 % and a mortality rate of 9.48 %, which has led to population aging, with 14.1 % of the population aged >65. Life expectancy is 76.01 years.

The National Integrated Health System (SNIS, *Sistema Nacional Integrado de Salud*) is composed of public and private health care providers who offer universal coverage with explicit guarantees [Comprehensive Health Care Plan (PIAS, *Plan Integral de Atención de Salud*)] whose funding comes from the National Health Fund (FONASA, *Fondo Nacional de Salud*) using a mixed mechanism of capitation and payment by results (health care goals). Furthermore, the system receives contributions from the national income. The high-cost and high-complexity coverage is implemented through a Public Fund (National Resource Fund). In Uruguay, government expenditure on health accounts for 6.93 % of the GDP. Leadership and governance of the health system come under the Ministry of Public Health, jointly with other dependent bodies.

### National stroke management plan in Uruguay

As agreed in the Ministerial Meeting of Gramado in 2018, where it was decided to carry forward a strong campaign to reduce the high impact of stroke in Latin America (8), a specific department responsible for devising the NSP was established within the MPH of Uruguay. The School of Medicine of the University of the Republic (UdelaR, *Universidad de la República*), the Medical Association of Uruguay, the National Academy of Medicine, scientific associations, and patient organizations were consulted. National and international experts from different fields were also consulted, including experts from the World Stroke Organization (WSO) and Global Stroke Alliance (GSA). After the different planning, development, writing, and correction stages, the first version of the National Stroke Protocol was published in Uruguay, in 2020 (9). It focused on three main verticals: prevention, stroke treatment (general management, reperfusion, stroke unit), and rehabilitation, including a specific protocol for pre-hospital management with a focus on baseline medical actions and key interventions of renowned efficacy to improve care provision times, such as pre-notification to the receiving hospital (10).

The measures included in the guidelines were assessed in relation to their budgetary financial impact by the Health Economy unit of the MoH, and the National Resource Fund. Presidential regulatory decrees and several ordinances were passed, which set the foundations of the legal framework for the NSP.

### Prevention

With the implementation of the NSP, massive campaigns related to prevention, risk factors, and early detection of stroke symptoms were launched on social and mass media. The Spanish acronym “ACV” was associated with the motto in Spanish “*actuemos con velocidad*” (let's act fast), following the model of other campaigns in Spanish for rapid detection of symptoms and activation of pre-hospital emergency services. In 2021, front-of-package food labeling started for products with excessive saturated fat, salt, sugars, and trans fatty acids, which was included by the WHO as a priority measure for the control of non-communicable diseases. Press and media coverage on the issue were undertaken across the country for 2 years. Furthermore, guidelines for the inclusion of these issues in the school curricula were established, and the implementation of the Hearts Program of the PAHO in Uruguay is currently being sought.

## Treatment

### Stroke as a health system goal

The “National Health Objectives 2030” of Uruguay established non-communicable diseases, including stroke, as a priority. In such a sense, one of the first measures was to position stroke as a “health care goal” within the whole Integrated Health System. Healthcare goals imply that healthcare providers are offered a financial incentive in return for compliance with the basic indices related to acute stroke treatment. In the first stage, healthcare providers must submit the “roadmap” (stroke code) followed by patients with stroke to the MPH. They need to establish how coordination with pre-hospital healthcare will be achieved, the organization of “stroke teams” in each institution, 24/7 coverage of CT technicians for rapid execution of scans, the existence of strict protocols for application of thrombolysis and thrombectomy procedures in each case, and the existence of stroke units, among others. On a quarterly basis, the “health care goals” become more complicated, accounting for the ratio of thrombolyzed patients, door-to-scan and door-to-needle times, and the disability measured as per the Rankin scale during follow-up. The quality of secondary prevention should also be considered. At present, we can affirm that 100% of healthcare providers in Uruguay, either public or private, have established their roadmaps in relation to stroke care. Preliminary outcomes show that since the onset of NSP, the average thrombolysis ratio has increased from 9 to 11%, as well as thrombectomy procedures, which have increased by 30%, compared to previously published national data (11). Some sites, such as Hospital de Clínicas (Public University Hospital) have shown substantial development, exceeding a 20% thrombolysis ratio in 2020 and 2021, and being awarded the Angel's Award Diamond Status of the WSO.

#### Stroke centers in Uruguay

Forty-two Stroke Ready Centers (SRC) and seven Comprehensive Stroke Centers (CSC) were recognized country-wide. Geographic distribution enables compliance with the international recommendation of, at least, one stroke unit per 100,00 inhabitants, and of one angiography suite per 1,000,000 inhabitants. Medical coverage relating to stroke is universal and free, establishing that the patient is provided with care in the closest stroke center, irrespective of the healthcare provider servicing the patient. At each center, a multidisciplinary “stroke team” was identified and created. Thrombolytic drugs are covered by the National Therapeutic Forum, ensuring universal coverage throughout the health system. Uruguay has a wide coverage of the pre-hospital system, assisted by medical staff, and with adequate distribution of tomography equipment. For the creation of the CSC, international experts offered their support, ensuring the existence of seven state-of-the-art angiography machines to perform thrombectomy procedures. Two were established in the North of Uruguay, and five in the South, where most of the population resides. A committee of experts validated the competencies of Uruguayan neuro-interventional surgeons, trained abroad at reference centers. Funding for thrombectomy procedures was established through the National Resource Fund (NRF), with a two million US dollar fund for the 1st year.

Uruguay complies with the certification of Stroke Centers of the WSO and the Iberoamerican Cerebrovascular Diseases Society (SIECV, *Sociedad Iberoamericana de Enfermedad Cerebrovascular*). A local Accreditation Committee was established, which promoted international certification of stroke centers with quality standards, having already achieved certification of local centers. The International Accreditation Committee recently visited several centers in the country. Hospital de Clínicas of Uruguay was certified as a CSC. Within this context, the integration of Uruguay into the international registries such as Safe Implementation of Treatments in Stroke (SITS) and Registry of Stroke Care Quality (RES-Q) was promoted.

### Medical education

The NSP was massively fostered, as well as its recommendations for hyperacute and prehospital management. Moreover, stroke training courses with different complexity levels were offered countrywide, following the Angel's Initiative model (with the endorsement of the European Stroke Organization - ESO) for all the interdisciplinary teams. Including both in-person and virtual training workshops, more than 1,500 healthcare providers were present. An augmented reality system was used for the resolution of case studies and *in situ* simulations were made in the different centers. The teaching staff was composed of experts from the School of Medicine, the MPH, scientific associations such as the Neurology Association, and international expert guests. Uruguay also joined the Mission Thrombectomy Campaign leadership of the WSO and GSA. Through this initiative, neuro-interventional surgeons who work in reference centers worldwide, came to Uruguay to carry out on-call shifts and procedures together with Uruguayan specialists, as well as to participate in case discussions, seminars, and academic activities.

### Rehabilitation

A national survey of neurological rehabilitation centers was established, as well as a guide of available geo-referenced resources for patients and their families and caregivers, who also have access to a care guide after a stroke (12). The Uruguayan government made important investments in rehabilitation centers at two public sites (Hospital de Clínicas and Banco de Seguros del Estado).

## Conclusions

Uruguay has demonstrated a strong commitment to addressing stroke by implementing the NSP which organized and optimized the health system resources for better and earlier treatment of stroke. The NSP was accompanied by an extensive prevention and community dissemination campaign as well as training of the healthcare staff. A significant regulatory framework was also established and the inclusion of healthcare goals enables quality control in establishing a continuous improvement process. From its initial stages, the NSP has shown signs of improvement in terms of access to recanalization therapies, in compliance with the main objective of public health, enabling universal access to stroke prevention, treatment, and rehabilitation, a highly-prevalent condition in the country having a deep individual, social and family impact on the health system and the Uruguayan society as a whole.

## Data availability statement

The original contributions presented in the study are included in the article/supplementary material, further inquiries can be directed to the corresponding author/s.

## Author contributions

IA: original idea, general coordination, review and co-writing of the entire article. AS: review of stroke care systems in the world, contribution to the introduction. AG: writing the section on establishing stroke centers. CC: writing on medical education. MM: contribution of statistical data. BT and SY: wrote about rehabilitation. RD and TM: wrote about certifying stroke centers. All authors contributed equally to the original idea of the article and its conclusions.

## References

[1] GBD Compare [Uruguay]. Healthdata.org. Available online at: https://vizhub.healthdata.org/gbd-compare/ (accessed October 10, 2022).

[2] Hackembruch HJ Perna A Ketzoian CN. Mortality trends by stroke in Uruguay. J Neurol Sci. (2013) 333:e209–10. 10.1016/j.jns.2013.07.834

[3] Ouriques Martins SC Sacks C Hacke W Brainin M de Assis Figueiredo F Marques Pontes-Neto O. Priorities to reduce the burden of stroke in Latin American countries. Lancet Neurol. (2019) 18:674–83. 10.1016/S1474-4422(19)30068-7 31029579

[4] Hochmann B Coelho J Segura J Galli M Ketzoian C Pebet M. The incidence of cerebrovascular accidents in the town of Rivera, Uruguay. Rev Neurol. (2006) 43:78–83. 10.33588/rn.4302.2005628 16838254

[5] Rodriguez MJ. Uruguay's Health Ministry (2022). [Unpublished Data].

[6] Camejo C Legnani C Gaye A Arcieri B Brumett F Castro L. Unidad de ACV en el Hospital de Clínicas: comportamiento clínico-epidemiológico de los pacientes con ACV (2007–2012). Arch Med Intern. (2015) 37:30–5. Available online at: http://www.scielo.edu.uy/scielo.php?script=sci_arttext&pid=S1688-423X2015000100006 (accessed July 31, 2022).

[7] Resultados del Censo de Población 2011: población, crecimiento y estructura por sexo y edad. Instituto Nacional de Estadísitca. Uruguay. Available online at: https://www.ine.gub.uy/censos-2011. (accessed October 10, 2022).

[8] Martins SCO Lavados P Secchi TL Brainin M Ameriso S Gongora-Rivera F. Fighting against stroke in Latin America: a joint effort of medical professional societies and governments. Front Neurol. (2021) 12:743732. 10.3389/fneur.2021.743732 34659101 PMC8517273

[9] Protocolo Nacional de ACV. 2020. Ministry of Public Health of Uruguay. (2022). Available online at: https://www.gub.uy/ministerio-salud-publica/comunicacion/publicaciones/protocolo-nacional-acv (accessed October 10, 2022).

[10] Savia A. Nuevas perspectivas en el manejo prehospitalario del accidente cerebrovascular. Neurol argent. (2020) 12:260–70. 10.1016/j.neuarg.2020.07.004

[11] Preve Cocco F Gaye A Hackembruch J. Cohort of patients with ischemic stroke - thrombolyzed and candidates for mechanical thrombectomy - from the Stroke Unit - Hospital de Clínicas (period March 2014-16) - Uruguay. Rev Uruguay Med Int. (2016) 1:35–43.

[12] Manual para pacientes y su familia luego de un ataque cerebrovascular (ACV). Uruguay. (2019). Available online at: https://www.gub.uy/ministerio-salud-publica/sites/ministerio-salud-publica/files/documentos/publicaciones/Manual%20cuidadores%20post%20ACV. (accessed October 10, 2022).

